# Supplementary material for: Effective Removal of Tetracycline from Water Using Copper Alginate @ Graphene Oxide with In-Situ Grown MOF-525 Composite: Synthesis, Characterization and Adsorption Mechanisms
Source: Nanomaterials (Basel). 2022 Aug 23;12(17):2897. doi: 10.3390/nano12172897 (PMC9458214; doi:10.3390/nano12172897)
Supplement: Supplementary file 1 [file nanomaterials-12-02897-s001.zip › nanomaterials-1861240-supplementary.pdf]

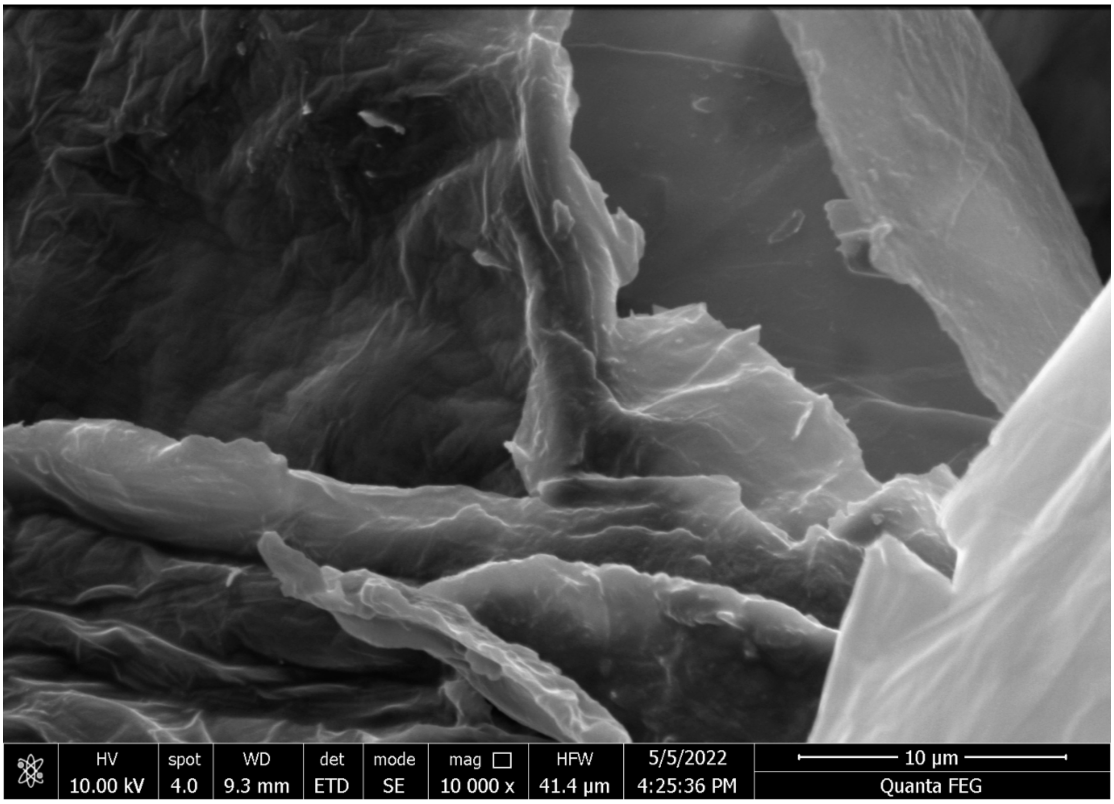

Figure S1. SEM micromorphology of GO

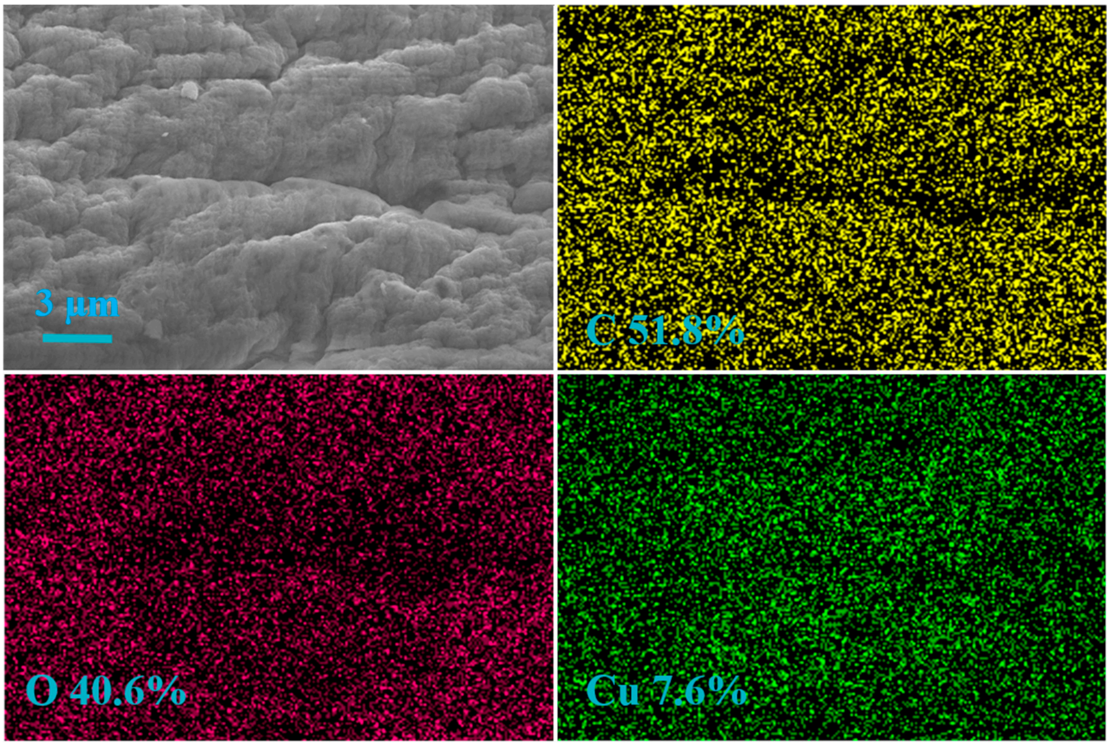

Figure S2. SEM images of Alg-Cu and the elemental distributions of C, O and Cu.

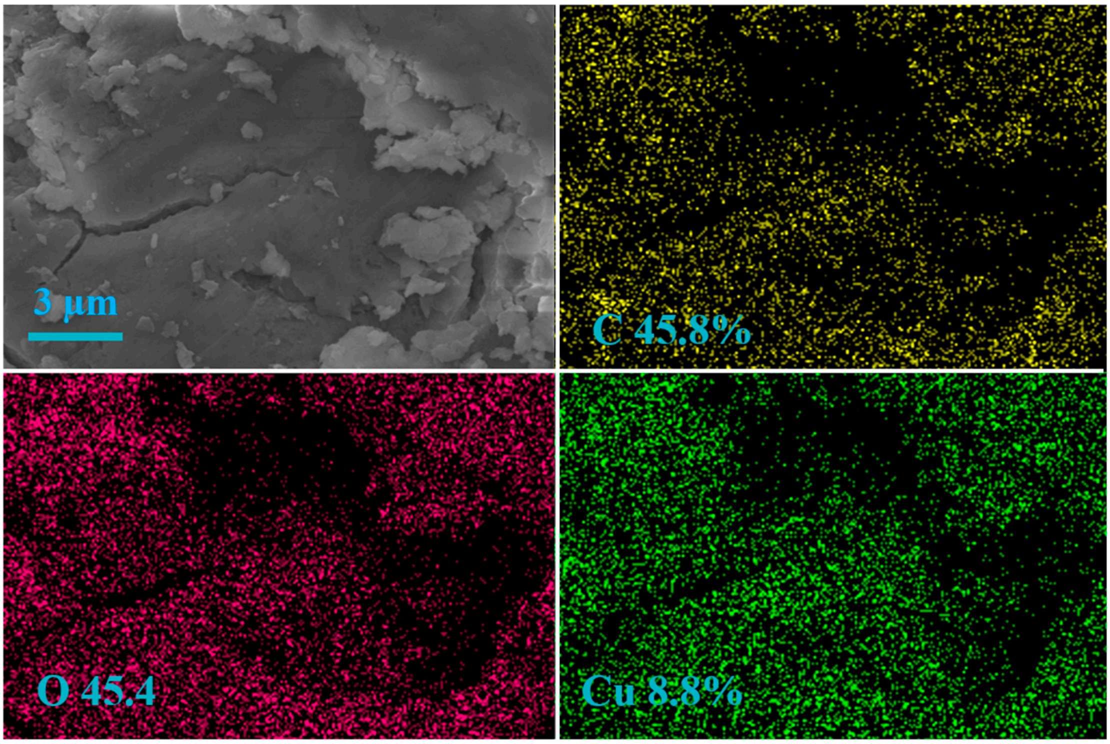

Figure S3. SEM images of Alg-Cu@GO and the elemental distributions of C, O and Cu.

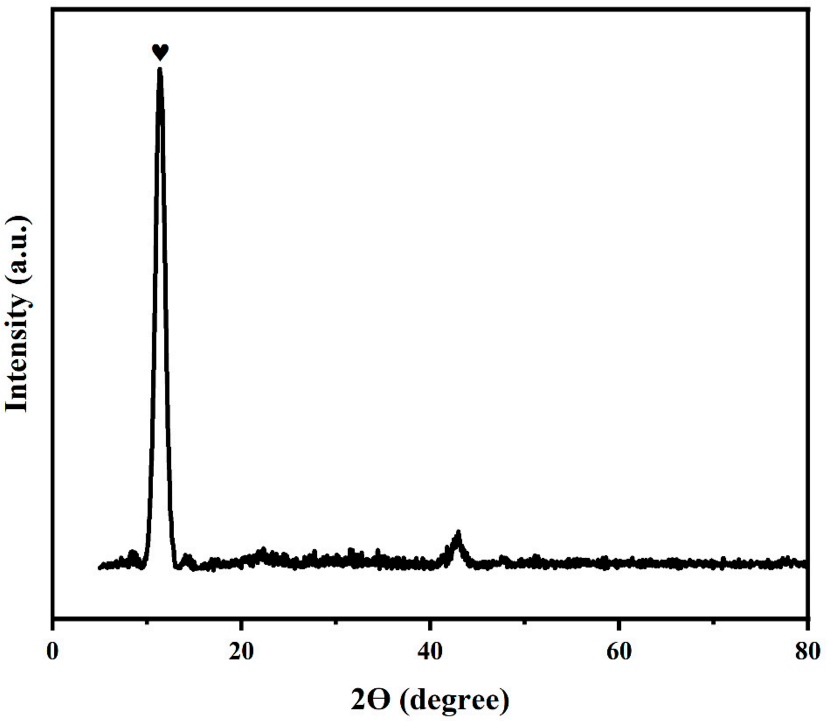

Figure S4. XRD patterns of GO.

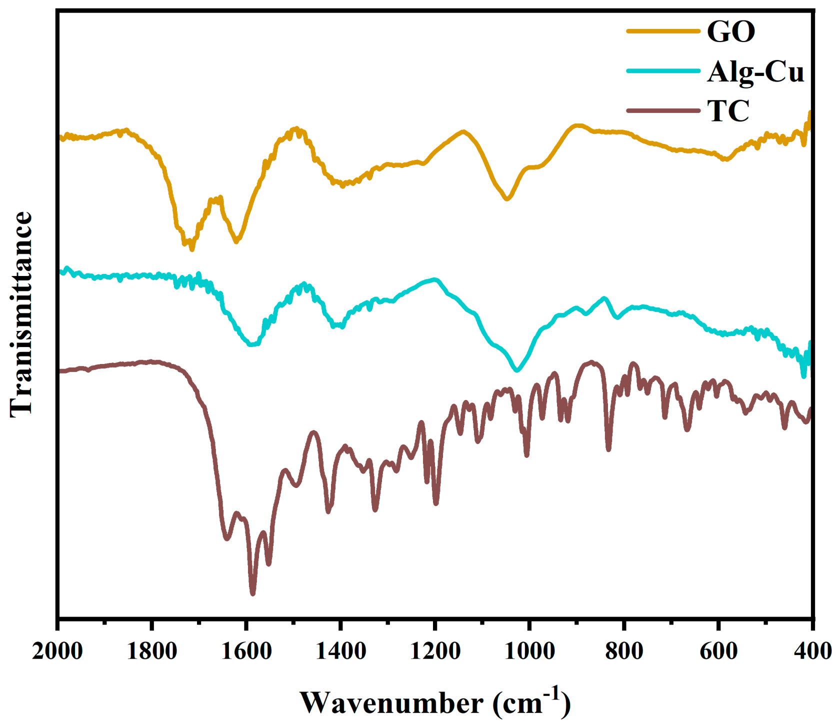

Figure S5. FTIR spectra of GO, Alg-Cu and TC.

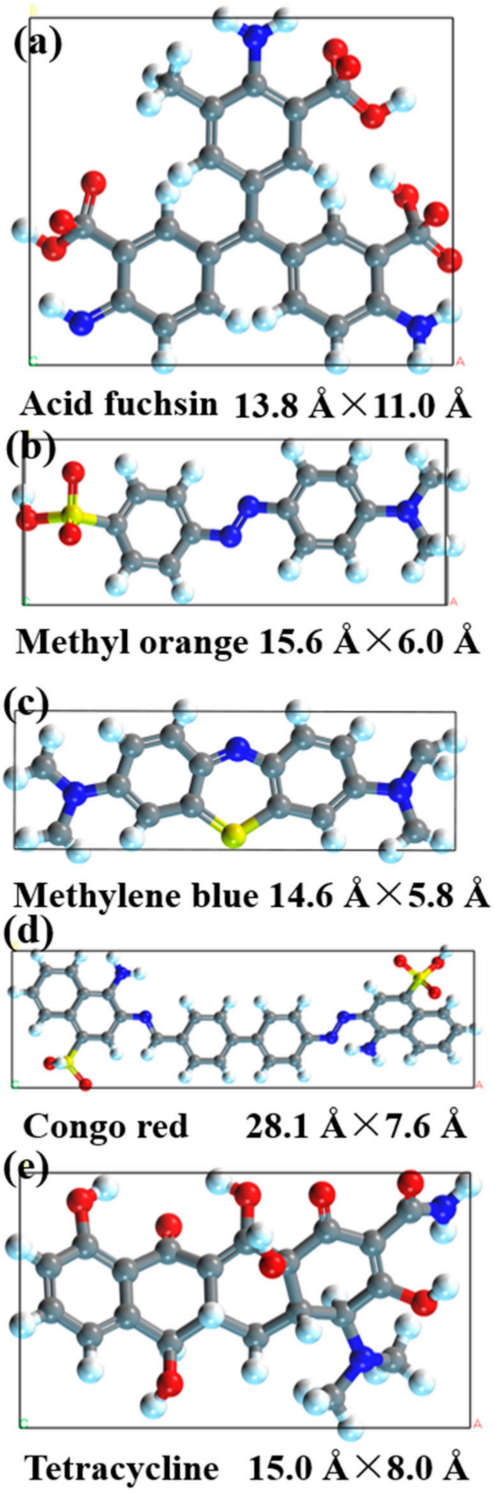

Figure S6. Molecular size of different pollutants optimized by Martials studio.
